# Supplementary material for: Stabilization of a molecular water oxidation catalyst on a dye−sensitized photoanode by a pyridyl anchor
Source: Nat Commun. 2020 Sep 14;11:4610. doi: 10.1038/s41467-020-18417-5 (PMC7490713; doi:10.1038/s41467-020-18417-5)
Supplement: Supplementary file 1 — Supplementary Information [file 41467_2020_18417_MOESM1_ESM.pdf]

Supplementary Information

Stabilization of a Molecular Water Oxidation Catalyst on a Dye-Sensitized Photoanode by Pyridyl Anchor

Yong et al.

Materials

All synthetic reactions were carried out under N_2 atmosphere with standard Schlenk techniques. According to standard methods, solvents were dried and purified prior to use. The compounds **RuP**$^{2+}$,[1] pyridyl-4-phosphonic acid[2] were prepared according to the literature procedures. NH_4PF_6($\geq$ 99.5%), ruthenium(III) chloride ($RuCl_3$, Ru content 45.0-55.0%), 4-bromopyridine hydrochloride ($\geq$ 98.0%), diethyl phosphite ($\geq$ 99.0%), 4,4'-bipyridine ($\geq$ 99.0%) were purchased from Aladdin. 4,4'-dibromo-2,2'-bipyridine ($\geq$ 97.0%), Sephadex LH-20 (BR) were purchased from Macklin Inc. All other chemicals are commercially available. High purity water (18.2 MΩ·cm) supplied by a Milli-Q system (Millipore, Direct-Q 3 UV) was used in all experiments. FTO substrates were purchased from Dalian Heptachroma SolarTech Co., Ltd. (thickness of ~2.2 mm, transmittance of > 90%, resistance < 15 Ω/cm2). Before using, the FTO substrates were ultrasonically cleaned in deionized water, ethanol and acetone, respectively.

Characterization

^{1}H NMR spectra were collected at 298 K using a Bruker DRX-500 instrument. Electrospray ionization mass spectra were recorded on a LTQ Orbitrap XL Micromass spectrometer (Thermo Scientific, USA). Electrochemical measurements were taken with a CHI 660E electrochemical potentiostat (Shanghai Chenhua, China). The (inductively coupled plasma mass spectrometry) ICP measurements were taken with Optima2000DV inductively coupled plasma mass spectrometry (PerkinElmer, USA).

Electron microscopy

Side-view Scanning electron microscopy (SEM) images of TiO_2 films electrode cross section was conducted with a Hitachi SU8220 instrument with an accelerating voltage of 5.0 kV. For Transmission Electron Microscopy (TEM) imaging, TiO_2 films was fabricated on FTO substrates and scraped off mechanically. The powders were re-dispersed in ethanol by sonication. A drop of each suspended sample was placed on a TEM grid and dried before being imaged. TEM images were obtained by Thermo Scientific TF30instrument.

Gas Chromatography Measurements

The PEC reaction was operated at a constant bias of 0.2 V *vs.* NHE under illumination (< 400 nm ,100 mW cm^{-2}) for 2 h. The evolved oxygen and hydrogen were quantified with a Techcomp 7890T gas chromatograph equipped with a thermal conductivity detector (TCD) and an Agilent 19095P-MS0 column. The oven temperature was 70℃, the temperatures for both injection port and thermal conductivity detector were set to 110℃. Argon was used as the carrier gas at a flow rate of 4 mL/min. Before the experiment, the electrolyte in the PEC device was thoroughly degassed by Argon. After the PEC reaction, 500 μL of the gas sample taken from the headspace of the sealed cell was injected into the gas chromatograph. The volume of the headspace in the PEC cell was measured and the molar amount of O_2 and H_2 was calculated based on the standard curve for each gas.

The standard curve for oxygen, fitted with the function $y = 3.15 \times 10^{-6} x$, was obtain by plotting the percentage content of oxygen in the headspace as a function of the peak area in GC. Similarly, a standard curve for hydrogen, fitted with the function $y = 3.31 \times 10^{-7} x + 1.10 \times 10^{-4}$, was obtained.

ICP measurements

RuP adsorbed on TiO_2 was desorbed by 1M NaOH and the eluent was diluted with H_2O into a 5 mL solution. The atomic emission spectroscopy of Ru was then analyzed by PerkinElmer, inductively coupled plasma mass spectrometry. The amount of Ru^{2+} in solution was finally quantified from a standard curve of atomic emission spectroscopy versus Ru^{2+} concentration.

Desorption Measurements

In a typical experiment, a catalyst decorated TiO_2 film was inserted at an angle of 45° into a standard UV-Vis cuvette filled with 3 mL acetate buffer solution (pH 5.8). As a prerequisite for surface dimerization, the oxidation of catalyst was inhibited by the addition of 2% sodium ascorbate into the solution. The desorption of catalyst from the film was monitored by recording the decay of the characteristic MLCT absorption at 480 nm every 2 minutes (Figure S14). The k_{obs} was obtained by fitting the decay kinetics with the biexponential function: $k_{obs} = A_1e^{-tk_1} + A_2e^{-tk_2}$

The multiexponential nature of the absorption changes under test conditions could

arise from a variety of competing decomposition pathways and local inhomogeneities in the metal-oxide surface structure. For comparative purposes, the results of the multiexponential analysis were represented by desorption rate constant, k_{des}, by calculating the weighted average lifetime: $1/k_{des} = <t> = \Sigma A_i k_i^2 / \Sigma A_i k_i$

Synthesis

Ru(bda)(4,4′-bpy)$_2$ (1): This complex was synthesized by a modified method reported in literature.[3] A mixture of 2,2'-bipyridine-6,6'-dicarboxylic acid (**bda**) (488 mg, 2.0 mmol), $Ru(DMSO)_4Cl_2$ (968 mg, 2.0 mmol), and Et_3N (1.6 mL) was refluxed in methanol (20 mL) for 4 h. After cooling to room temperature, the formed precipitate was filtered and washed with acetone and ether to get a reddish-brown powder of $Ru(bda)(DMSO)_2Cl_2$.

A 40 mL methanol solution containing $Ru(bda)(DMSO)_2Cl_2$ (500 mg, 1.0 mM) and 4,4'-bipyridine (**4,4′-bpy** 468 mg, 3.0 mmol) was refluxed for 4 h. The solvent was removed under reduced pressure, the residue was purified by silica gel chromatography with dichloromethane-methanol (20:1 to 1:1, V:V) as the eluent. Complex **1** was obtained as a dark red solid. Yield: 297 mg (40%) ^{1}H NMR (500 MHz, DMSO) δ 8.75 (s, 1H), 8.67 (d, 2H), 7.92 (d, 4H), 7.76–7.62 (m, 4H). ESI-MS: m/z = 657.20 $[M + H]^+$ (calcd: 657.07).

Ru(bda)(4-pyPO$_3$H)$_2$ (2): The same procedure as described above was employed for complex **2** except replacing 4,4'-bipyridine with pyridyl-4-phosphonic acid (**4-py**, 477 mg, 3.0 mmol). The crude product was purified by a Sephadex LH-20 column chromatography using H_2O as the mobile phase. The mobile phase is only propelled by gravity. Complex **2** was obtained as a dark red solid. Yield: 156 mg (20%). ^{1}H NMR (500 MHz, D_2O with ascorbic acid) δ 8.40 (d, 2H), 7.85–7.77 (m, 4H), 7.70 (d, 4H), 7.24 (dd, 6.0 Hz, 4H). ESI-MS: m/z = 329.94 $[M\text{-}2H]^{2-}$(calcd:329.97).

Ru(bda)(4-pyO(CH$_2$CH$_2$)$_5$PO$_3$H)$_2$ (3): This complex was synthesized by method reported in literature.[4]

Supplementary Figures

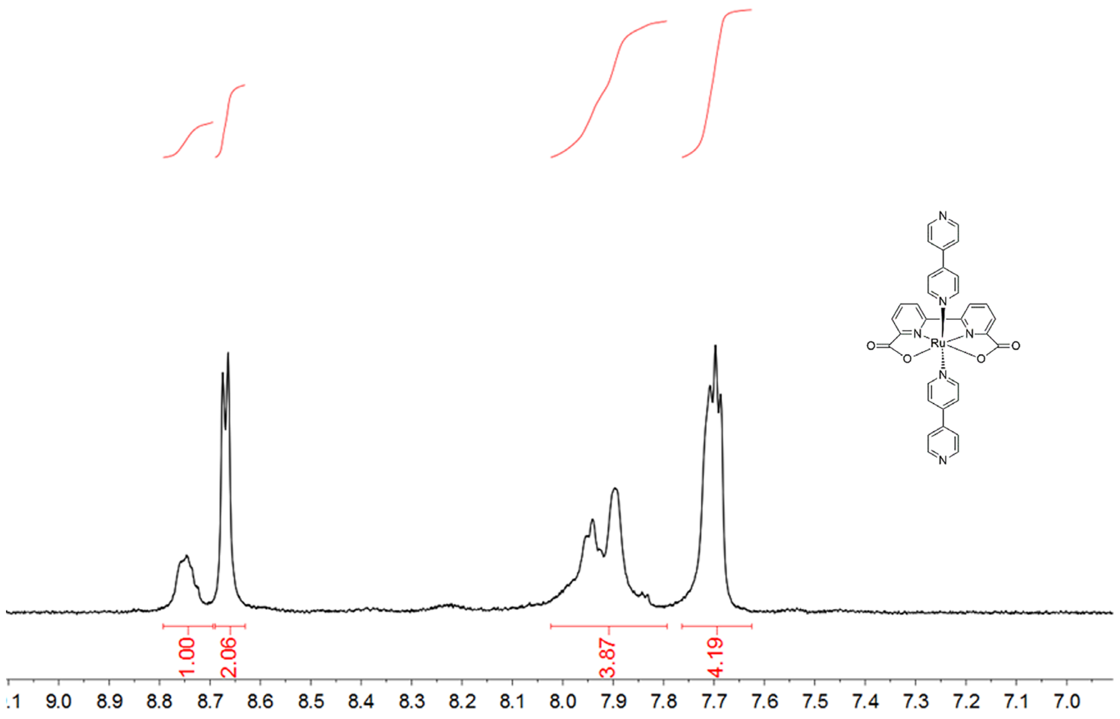

Supplementary Fig. 1 ^{1}H NMR spectrum of complex **1** in DMSO-d6.

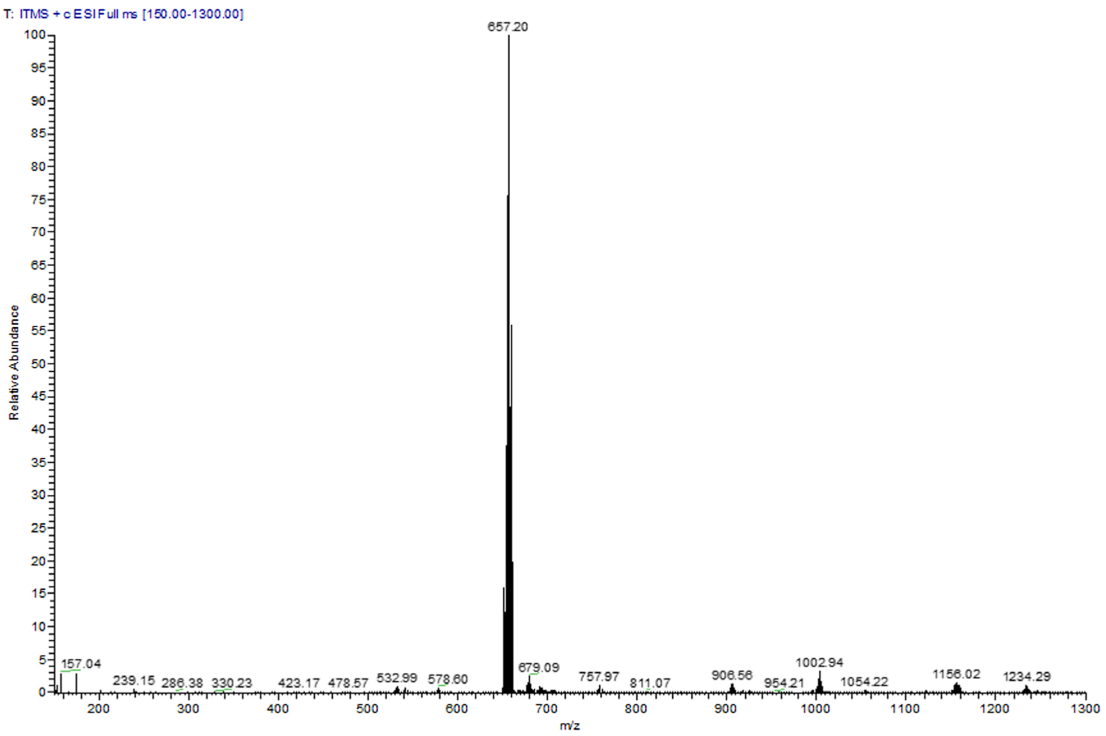

Supplementary Fig.2 ESI-MS spectrum of complex **1** in CH_3OH.

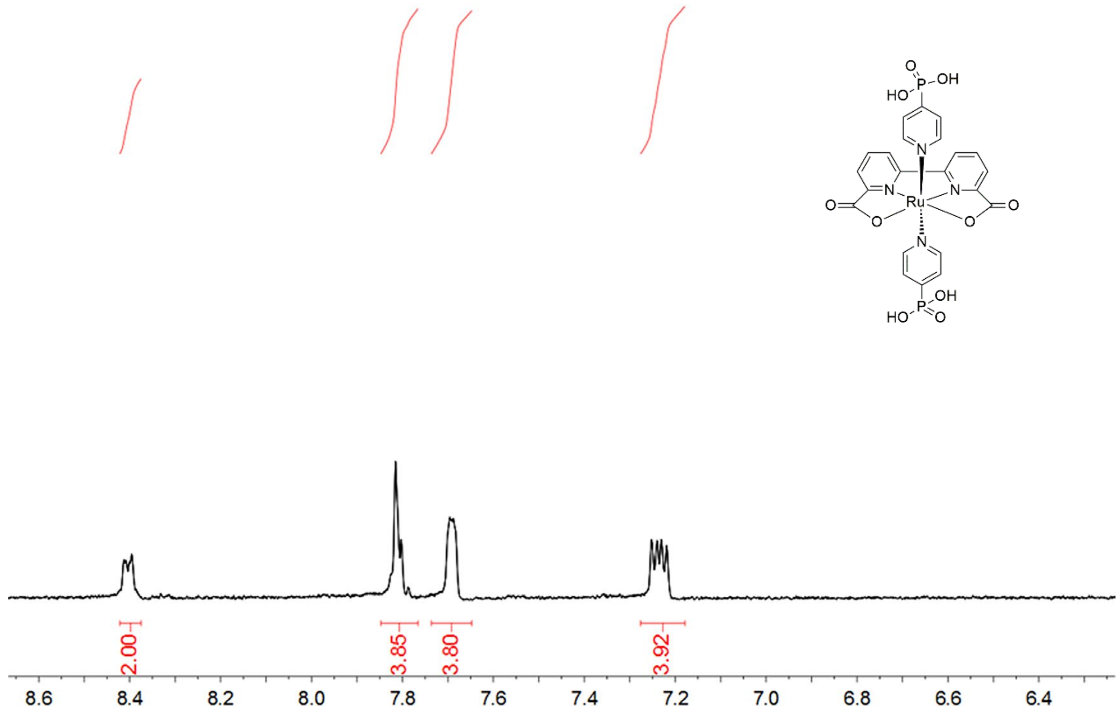

Supplementary Fig.3 ^{1}H NMR spectrum of complex **2** in D_2O with ascorbic acid.

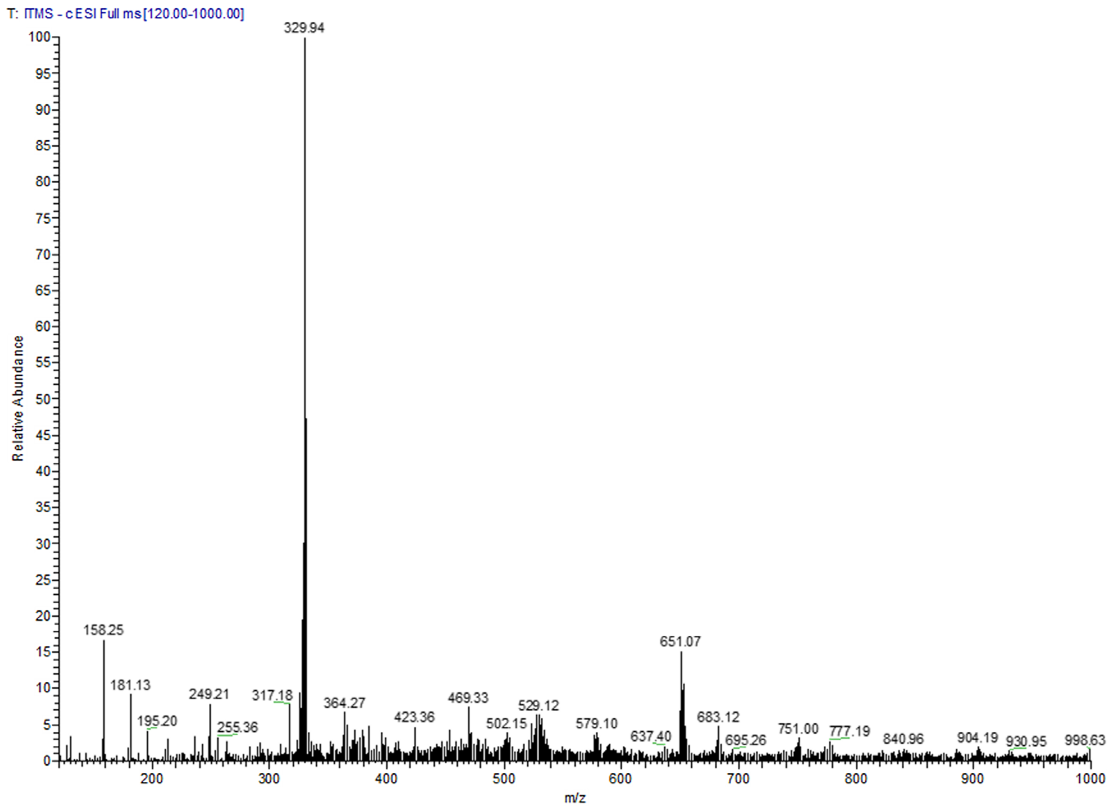

Supplementary Fig.4 ESI-MS spectrum of complex **2** in CH_3OH.

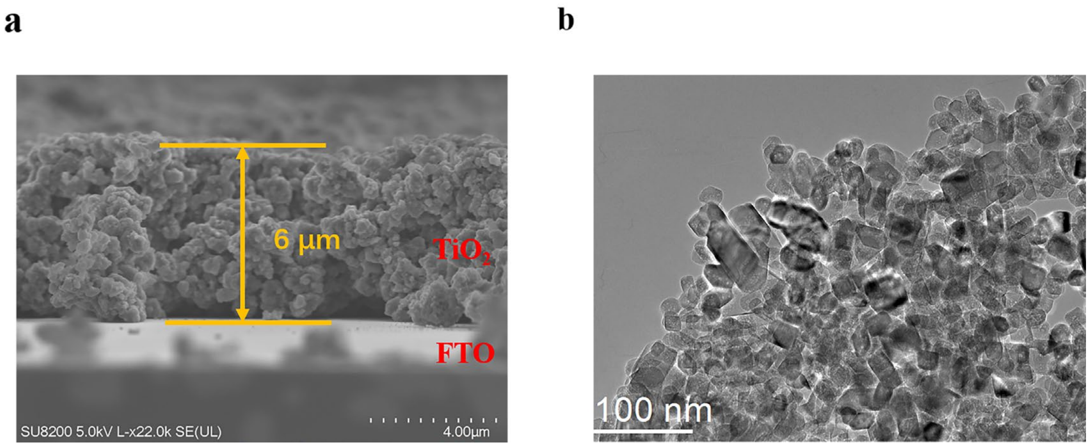

Supplementary Fig.5 (**a**) Side-view SEM images of electrode cross section, (**b**) TEM image of TiO_2 nanoparticles.

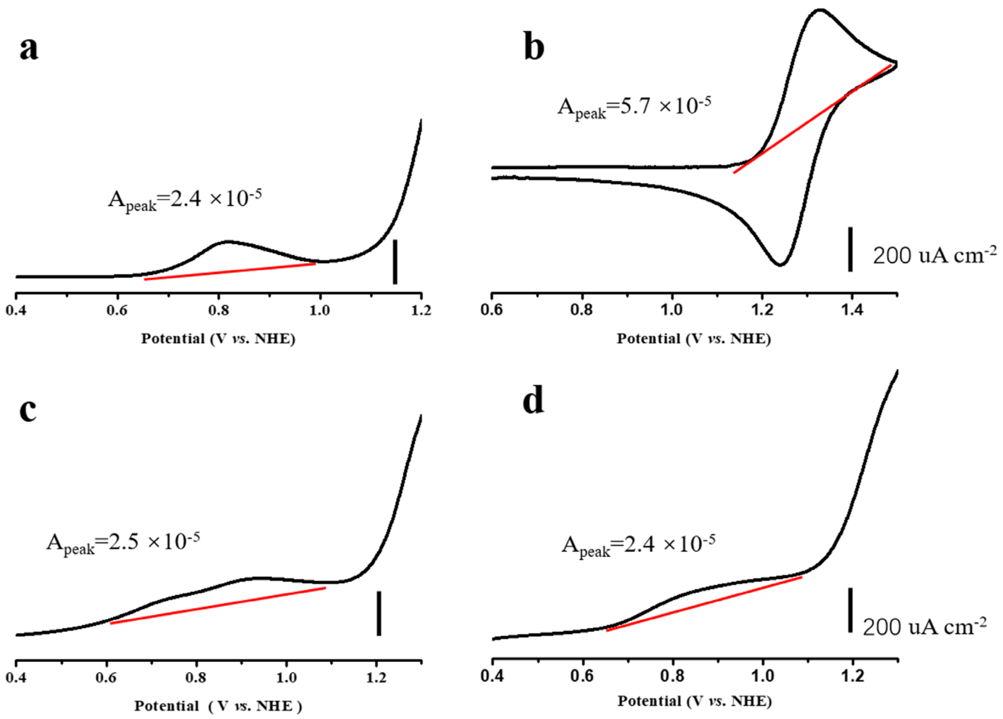

Supplementary Fig.6 Analysis of peak currents of TiO_2|-**RuP**$^{2+}$,**1** (**a**), TiO_2|-**RuP**$^{2+}$ (**b**), TiO_2|-**RuP**$^{2+}$,**2** (**c**) and TiO_2|-**RuP**$^{2+}$,**3** (**d**) in 0.1 M acetic acid/acetate buffer at pH 5.8 containing 0.5 M $NaClO_4$ at a scan rate 10 mV s^{-1}. Surface coverage (Γ) is calculated by integrating current peak using formula: $\Gamma = Q/nFA$ Where n = 2 or 1, A = 1 cm^2, F = 96500 C mol^{-1}, Q = A_{peak} / υ.

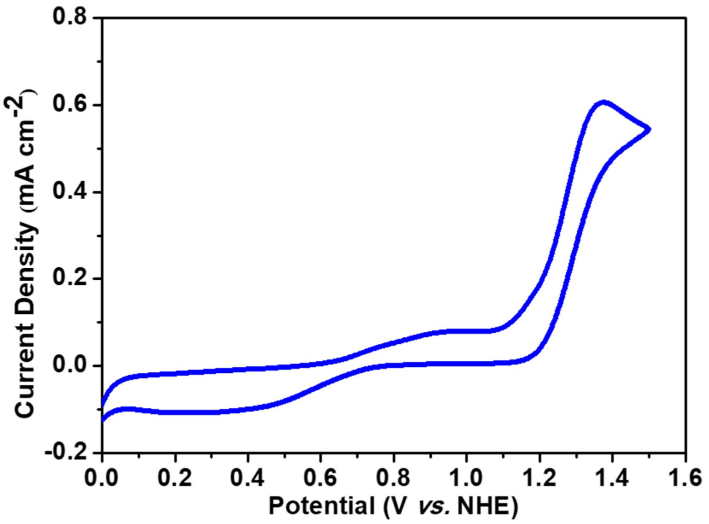

Supplementary Fig.7 Cyclic voltammogram of TiO_2|-**RuP^{2+}**,**2** and TiO_2 electrode in 0.1 M acetic acid/acetate buffer at pH 5.8 containing 0.5 M $NaClO_4$, scan rate 10 mV s^{-1}.

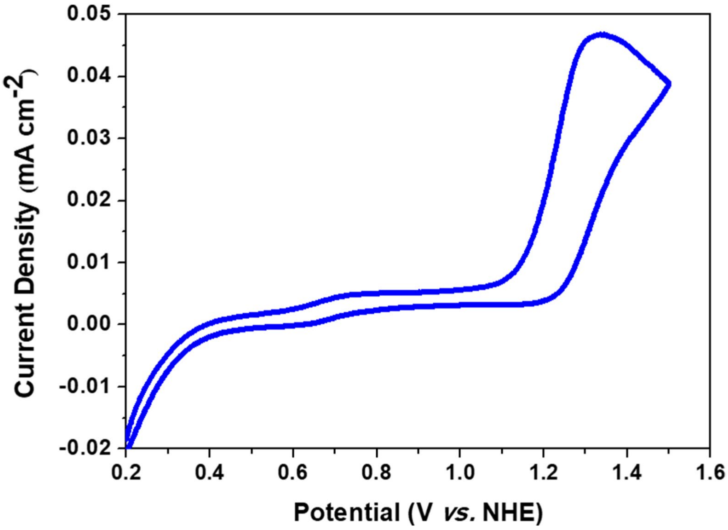

Supplementary Fig. 8 Cyclic voltammogram of TiO_2|-**RuP**$^{2+}$,**3** electrode in 0.1 M acetic acid/acetate buffer at pH 5.8 containing 0.5 M $NaClO_4$, scan rate 10 mV s^{-1}.

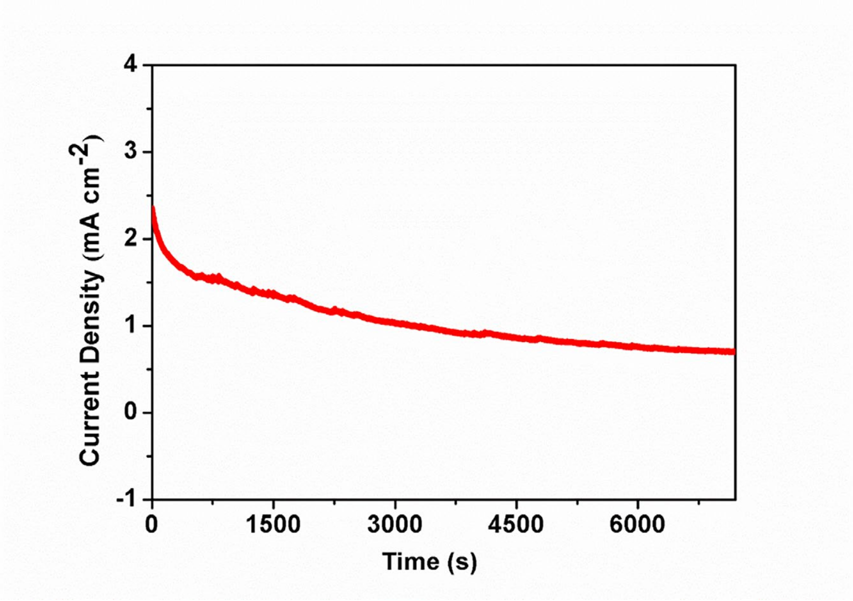

Supplementary Fig. 9 Current density-time traces of TiO_2|-**RuP**$^{2+}$,**1** electrode under illumination (> 400nm, 100 mW cm^{-2}) in 0.1 M acetic acid/acetate buffer at pH 5.8 containing 0.5 M $NaClO_4$.

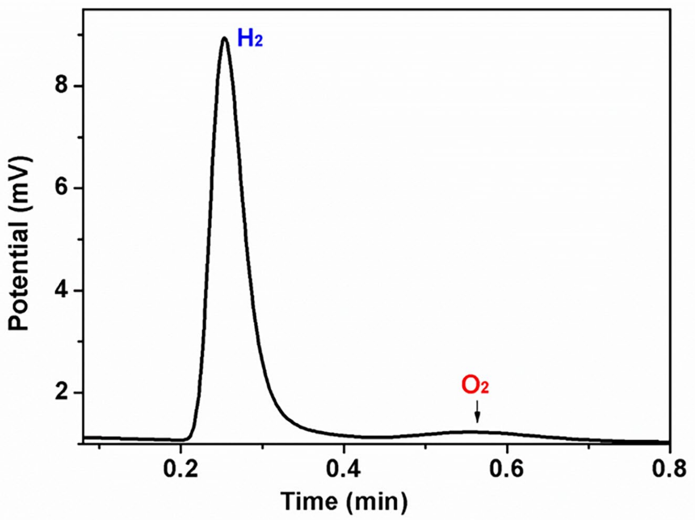

Supplementary Fig. 10 GC data for TiO_2|-**RuP**$^{2+}$, **1** electrode after light illumination. The molar amount of hydrogen was determined to be 6.24 μmol and the amount of oxygen was determined to be 12.7μmol.

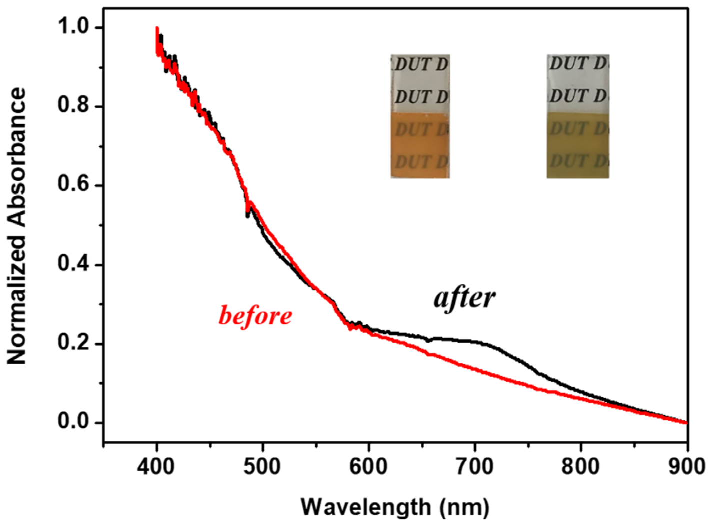

Supplementary Fig.11 UV-Vis spectra changes of TiO_2|-RuP^{2+},**3** before and after PEC experiments.

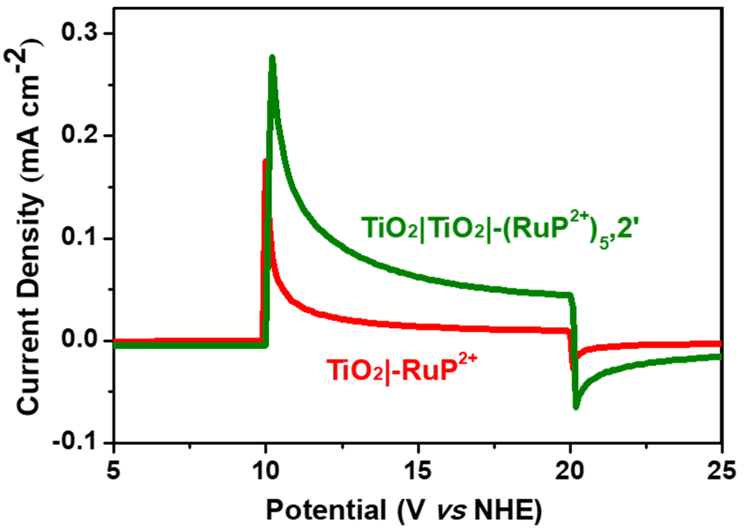

Supplementary Fig.12 Current density-time traces for TiO_2|-**RuP^{2+},2’** and TiO_2|-**RuP^{2+}** at a constant bias of 0.2 V *vs.* NHE with illumination at > 400 nm at 100 mW cm^{-2} at pH 5.8 in a 0.1 M acetate buffer containing 0.5 M $NaClO_4$.

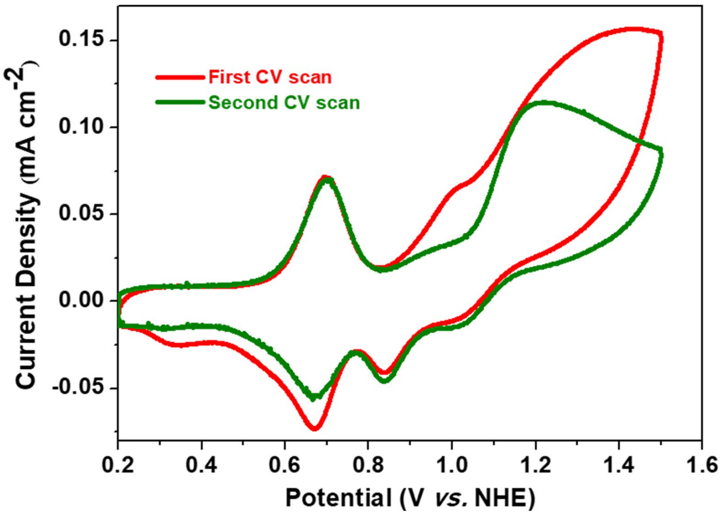

Supplementary Fig.13 Successive cyclic voltammograms for *nano*ITO|**3** in acetate buffer (pH 5.8, 0.5 M $NaClO_4$) at a scan rate 10 mV s^{-1}. The low current density is due to the high resistence caused by the long alkyl chain of **3**.

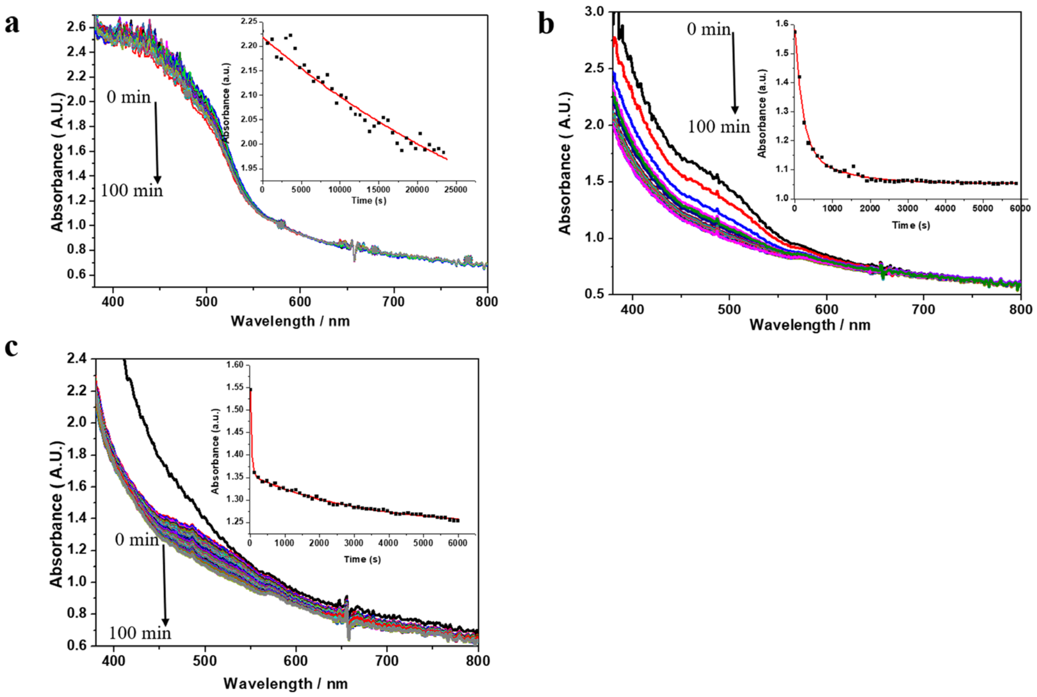

Supplementary Fig.14 Changes in the absorption spectra of TiO_2|-**1** (a), TiO_2|-**2** (b) and TiO_2|-**3** (c) in pH 5.8 acetate buffer containing 2% sodium ascorbate. Inset: absorbance-time trace at 480 nm.

Supplementary References

1 Norris, M. R. *et al.* Synthesis of Phosphonic Acid Derivatized Bipyridine Ligands and Their Ruthenium Complexes. *Inorg. Chem.*, **52**, 12492–12501 (2013).

2 Konar, S., Zoń, J., Prosvirin, A. V.. Dunbar, K. R. & Clearfield, A. Synthesis and Characterization of Four Metal-Organophosphonates with One-, Two-, and Three-Dimensional Structures. *Inorg. Chem.*, **46**, 5229–5236 (2007).

3 Jiang, Y., Li, F., Huang, F., Zhang, B. & Sun, L. Chemical and Photocatalytic Water Oxidation by Mononuclear Ru Catalysts. *Chinese J. Catal.*, **34**, 1489–1495 (2013).

4 Wang, D. *et al.*Interfacial Deposition of Ru(II) Bipyridine-Dicarboxylate Complexes by Ligand Substitution for Applications in Water Oxidation Catalysis. *J. Am. Chem. Soc.*, **140**, 719–726 (2018).
